# A Natural Language Processing–Based Virtual Patient Simulator and Intelligent Tutoring System for the Clinical Diagnostic Process: Simulator Development and Case Study

**DOI:** 10.2196/24073

**Published:** 2021-04-09

**Authors:** Raffaello Furlan, Mauro Gatti, Roberto Menè, Dana Shiffer, Chiara Marchiori, Alessandro Giaj Levra, Vincenzo Saturnino, Enrico Brunetta, Franca Dipaola

**Affiliations:** 1 Department of Biomedical Sciences Humanitas University Pieve Emanuele, Milan Italy; 2 Internal Medicine Humanitas Clinical and Research Center, IRCCS Rozzano, Milan Italy; 3 Active Intelligence Center IBM Bologna Italy; 4 IBM Research Zurich Switzerland

**Keywords:** COVID-19, intelligent tutoring system, virtual patient simulator, natural language processing, artificial intelligence, clinical diagnostic reasoning

## Abstract

**Background:**

Shortage of human resources, increasing educational costs, and the need to keep social distances in response to the COVID-19 worldwide outbreak have prompted the necessity of clinical training methods designed for distance learning. Virtual patient simulators (VPSs) may partially meet these needs. Natural language processing (NLP) and intelligent tutoring systems (ITSs) may further enhance the educational impact of these simulators.

**Objective:**

The goal of this study was to develop a VPS for clinical diagnostic reasoning that integrates interaction in natural language and an ITS. We also aimed to provide preliminary results of a short-term learning test administered on undergraduate students after use of the simulator.

**Methods:**

We trained a Siamese long short-term memory network for anamnesis and NLP algorithms combined with Systematized Nomenclature of Medicine (SNOMED) ontology for diagnostic hypothesis generation. The ITS was structured on the concepts of knowledge, assessment, and learner models. To assess short-term learning changes, 15 undergraduate medical students underwent two identical tests, composed of multiple-choice questions, before and after performing a simulation by the virtual simulator. The test was made up of 22 questions; 11 of these were core questions that were specifically designed to evaluate clinical knowledge related to the simulated case.

**Results:**

We developed a VPS called Hepius that allows students to gather clinical information from the patient’s medical history, physical exam, and investigations and allows them to formulate a differential diagnosis by using natural language. Hepius is also an ITS that provides real-time step-by-step feedback to the student and suggests specific topics the student has to review to fill in potential knowledge gaps. Results from the short-term learning test showed an increase in both mean test score (*P*<.001) and mean score for core questions (*P*<.001) when comparing presimulation and postsimulation performance.

**Conclusions:**

By combining ITS and NLP technologies, Hepius may provide medical undergraduate students with a learning tool for training them in diagnostic reasoning. This may be particularly useful in a setting where students have restricted access to clinical wards, as is happening during the COVID-19 pandemic in many countries worldwide.

## Introduction

Learning clinical diagnostic reasoning is a critical challenge for medical students, as fallacies in diagnostic reasoning may lead to patient mistreatment with negative consequences on patient health and health care costs [1]. Adequate training and coaching are pivotal aspects for the proper development of diagnostic skills. In medical schools, clinical coaching is currently performed under the direct supervision of senior doctors, mostly in the wards [2].

Constraints in human resources and increases in educational costs prompted the development of sustainable systems for optimizing medical student tutoring [3]. In addition, the strict need to keep social distances due to the recent COVID-19 worldwide outbreak has resulted in the temporary closure of universities in many countries and denied medical students from accessing clinical wards [4,5]. From an educational standpoint, this promotes the need for clinical training methods that do not require bedside didactic activities and that do not necessarily entail continuous direct supervision by experienced doctors [6,7]. Examples of these methods are simulators, which were developed not only to support learning of specific medical procedures, such as laparoscopy [8], but also to train students in clinical diagnostic reasoning as with virtual patient simulators (VPSs) [9]. A VPS is a computer program that simulates real-life clinical scenarios, enabling students to emulate the role of a doctor by obtaining a medical history, performing a physical exam, and making diagnostic and therapeutic decisions [10]. These computer-based simulators may complement traditional training techniques without requiring direct ward attendance [11].

Previous studies based on intelligent tutoring systems (ITSs) [12] have shown the effectiveness of programs [13] specifically developed to teach and practice knowledge in several areas, including mathematics and physics [14]. ITS technologies can be adapted to students’ specific learning needs, thus potentially increasing the simulator’s teaching effectiveness [15-17]. Natural language processing (NLP) may complement and support medical education techniques [18], particularly where the diagnostic reasoning aspect is concerned [15,19-22]. Notably, the combined use of NLP and ITS technologies in the simulation of virtual patients might promote students’ learning by making the student-software interaction more similar to a real-life scenario, while simultaneously giving the student appropriate feedback after every simulated medical activity.

The primary aim of this study was to develop a VPS that combines interactions in natural language and ITS components, in order to set up a tool that would enable students to improve their clinical diagnostic reasoning skills. A secondary aim was to preliminarily assess the short-term potential changes in medical knowledge of a group of undergraduate students after the use of the VPS.

This article is structured with the Methods section describing the architecture and main development features of the program and with the Results section describing both the program’s flow of use and the preliminary findings of a test performed on a population of undergraduate medical students.

## Methods

The program we developed is named Hepius, after the Greek god of medicine, and it is structured to perform as both a VPS and an ITS.

### Program Architecture

**Figure 1:**
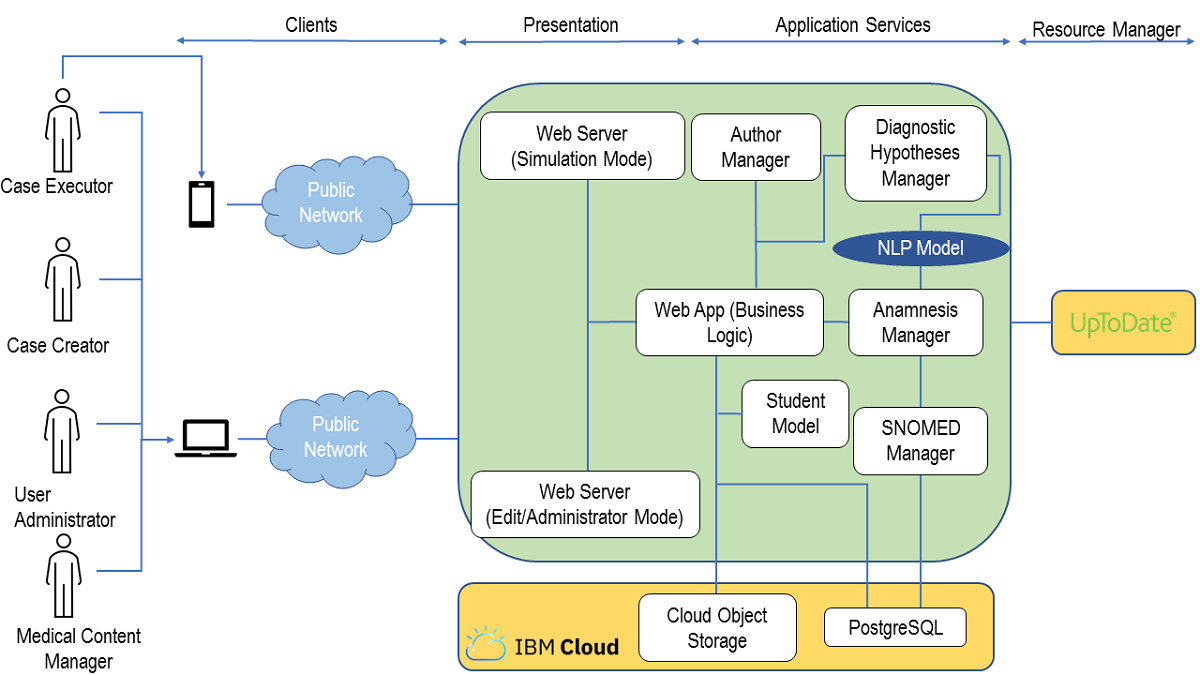
Overview of the Hepius program architecture. NLP: natural language processing; SNOMED: Systematized Nomenclature of Medicine; SQL: Structured Query Language.

The Hepius program architecture is outlined in Figure 1. Hepius has been designed and developed for four main categories of users: students, teachers, administrators, and medical content managers. The program is accessible through two main user interfaces: (1) a *mobile app*, developed using the Ionic Angular framework [23], that can be used to execute simulations and (2) a *web application*, developed using the PrimeFaces framework [24], that can be used to create and modify simulations or administer the system. Both user interface programs consume back-end services using representational state transfer application programming interfaces [25].

The Hepius back end has been developed according to the principles of microservices architecture [26] and it runs on the Cloud Foundry platform as a service (IBM Corp) [27]. The back-end components have been developed using three different programming languages: Java 8 (Oracle Corporation) as the main programming language, Python 3.7 (Python Software Foundation) for NLP services, and R 4.0 (The R Foundation) for the learner model.

The back end consumes an UpToDate service that is used to provide students with feedback. The Cloud Object Storage (IBM Corp) service is used as storage for multimedia files, whereas the PostgreSQL (Structured Query Language) (Compose) service is used as the main database. Both are provided in software-as-a-service mode by IBM Cloud.

### Natural Language Processing Algorithms

Interaction in natural language between the student and the program was developed for anamnesis, physical exams, medical test requests, and diagnostic hypothesis generation. Here we present, in detail, the diagnostic hypothesis generation and anamnesis modules.

#### Diagnostic Hypothesis Generation

When creating the simulation, the author decides which diagnostic hypotheses may be reasonable for the clinical case (ie, reference hypotheses). When the student formulates a diagnostic hypothesis in free text, Hepius assesses its correctness by calculating the Systematized Nomenclature of Medicine (SNOMED) graph path distance (ie, the minimum number of edges in any path connecting the two nodes) between the student’s diagnostic hypothesis and all the reference hypotheses. If any of the reference hypotheses have zero distance from the student’s hypothesis, then the student’s hypothesis is marked as correct and is inserted into the differential diagnosis. Should the distance be greater than 5, the hypothesis is considered incorrect. Whenever the distance is between 1 and 4, the hypothesis is considered to be close to the correct one and the student is provided with feedback that points toward the closest reference hypothesis.

To find the best match between the input text string and the concepts in SNOMED ontology, we used Jaccard similarity [28] between token lists obtained from texts associated with concepts, including synonyms, after removal of stop words.

The entire diagnostic hypothesis module is implemented using only open-source code. The programming language is Python 3.7; the main libraries are Medical Terminologies for Python (PyMedTermino) [29], for interaction with the SNOMED CT (Clinical Terms) database, and Natural Language Toolkit (NLTK) 3.5 [30], for basic NLP operations (eg, tokenization).

#### Anamnesis

When the student formulates an anamnestic question, it is matched to the most semantically similar one present in the list of reference questions created by the teacher. The estimation of the semantic similarity of two sentences cannot simply be reduced to the semantic similarity of tokens inside the sentence (eg, using an ontology) because the meaning of a sentence depends on its extremely variable syntactic structure.

**Figure 2:**
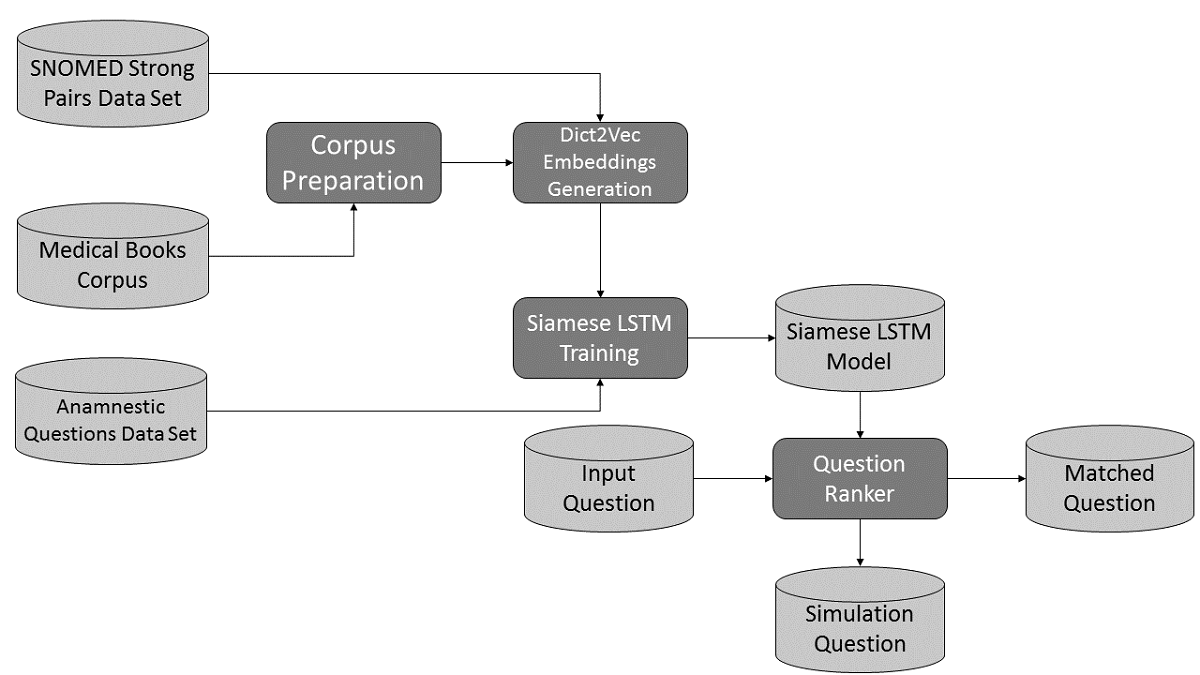
Pipeline of the history-taking natural language processing algorithms. Light grey cylinders identify data sources and dark grey boxes identify algorithms. LSTM: long short-term memory; SNOMED: Systematized Nomenclature of Medicine.

This *question matching* problem [31] has been addressed by developing an ad hoc pipeline of NLP algorithms (see Figure 2). The pipeline is based on a Siamese long short-term memory (SLSTM) network [32], trained on 7000 pairs of semantically equivalent and inequivalent anamnestic questions, that provides a probabilistic estimate of the semantic equivalence. This estimate is then used to rank all the reference anamnestic questions, thereby enabling the identification of the most similar one.

The SLSTM network requires a *word embedding* function [33] that converts words into tuples of real numbers (ie, vector representation) in such a way that semantically close words are transformed into vectors that are close according to a vector space metric [34]. Among the available unsupervised algorithms that learn word embedding, we decided to test Word2vec [35,36], Doc2vec [37], and fastText [38]. For all models, we generated our own embedding in an unsupervised way by means of the gensim library [39] using a corpus of medical textbooks and compared the overall pipeline performance with pretrained word embedding.

Using the medical textbooks corpus, the fastText word embedding that was generated proved to be superior in our setting compared to the other models, but it was still unable to correctly embed relevant pairs of medical synonyms. This problem has been addressed by the use of Dict2vec [40], introducing a form of weak supervision.

Long short-term memory (LSTM) networks [41] are neural networks that, like recurrent neural networks [41], can handle input sequences of arbitrary length by reusing at each computation step the same set of parameters, thereby reducing model complexity. LSTM networks are commonly used to tame the intrinsic instability of recurrent neural networks due to exploding and vanishing gradients [41]. Unlike more recent models, such as the Transformer [42], they are not designed for parallel computation being based on sequential inputs. In our context, we have two different inputs (ie, questions) that need to be compared; as a consequence, we need two LSTM networks that elaborate the inputs in parallel. For this purpose, we used SLSTM networks, whose key characteristic is that the two LSTM networks have exactly the same weights. The outputs of the networks are then compared using Manhattan distance [32].

The *question ranker* uses the trained SLSTM network model to compare the student input question with all the reference questions present in the simulation and ranks them according to the model output probability. A fixed probability threshold is used to decide whether the program should return a single question, multiple questions, or no questions. Returning multiple reference questions is undesirable because the program would be helping students in identifying reference questions that the student has not yet conceived, in contradiction with the didactical objective of having the student figure out the correct questions. On the other hand, returning matched questions only when the probability is very high could frustrate the students who would not receive correct semantic matches due to the fact that the algorithm has assigned low scores to these matches. The didactical decision we took was to fix a threshold and return all questions whose probability exceeds that threshold up to a maximum of three questions.

The anamnestic questions module is entirely written using open-source libraries to foster reproducibility. The programming language used to develop the module is Python 3.7. To generate the word embeddings, we used Dict2vec, for the reasons previously explained, by using the C code made available by the Dict2vect creators [43]. SLSTM networks were implemented using TensorFlow [44] and Keras [45]. The rationale underpinning the use of the SLSTM network is provided above; in addition, see Mueller and Thyagarajan [32] and Chen et al [46] for further details. An example of implementation strategy was found in Park [47]. The scikit-learn library [48] was used for basic data manipulations (eg, stratified train-test split). For basic NLP tasks (eg, tokenization and stemming), we used NLTK [29].

To test the above algorithms, we have developed six test sets, built out of six different simulations, with a total number of 547 questions, and measured the overall question matching accuracy. We obtained an accuracy greater than 70% for rank 1 matches and greater than 80% for rank 3 matches, as summarized in Table S1 of Multimedia Appendix 1.

### Intelligent Tutoring System Development

ITSs are based on the concepts of an *inner loop* (ie, step-by-step feedback and hints during the execution of the learning unit) and an *outer loop* (ie, indications of what is the optimal next learning step) [49]. Out of the five key models of an ITS, in Hepius we implemented the following three: (1) the *domain model*, a decomposition of the knowledge corpus into concepts to be taught; (2) the *assessment model*, the definition of tests aimed at assessing the level of the student’s understanding; and (3) the *learner model*, a mathematical model to predict learners’ results when compared with assessments.

In Hepius, the *domain model* knowledge units are the diagnostic hypotheses (ie, diseases) and the diagnostic factors (ie, signs, symptoms, physical findings, and medical tests).

The Hepius *assessment model* works by comparing every student’s action with the reference list containing all the possible correct actions written by the creator of the clinical case.

The Hepius *learner model* is a Bayesian Knowledge Tracing algorithm [50,51] that takes as an input the student performance in the execution of the binary analysis, for any diagnostic hypothesis, across multiple simulations. Bayesian Knowledge Tracing is based on a hidden Markov model (HMM) that provides an estimate of the probability that a student has a skill—in our context, the clinical understanding of a disease or diagnostic hypothesis—given his or her learning history—in our context, the results obtained during the analysis of the disease in previous simulations. To implement the algorithm, we used R packages HMM [52] and seqHMM [53].

### Short-term Learning Test Protocol

A total of 15 medical students attending their fifth year at the Humanitas University Medical School in Italy participated in the test. Students were already acquainted with Hepius, as they had received specific introductory lectures and used them to perform simulated clinical cases in the preceding weeks.

The 2-hour-long test was conducted in the Humanitas University computer room, where students used individual desktop computers. On the day of the test, all students began by taking a uniform presimulation written test, made up of 22 multiple-choice questions (see Multimedia Appendix 2), to assess their baseline knowledge on chest pain and shortness of breath. The test topics had been previously covered during the semester. Each question was worth 1 point. Among the 22 questions, there were 11 *core* questions, presented in random order, which had been specifically designed to evaluate the knowledge that could be acquired directly by performing the simulation with Hepius. Thereafter, the students had 60 minutes to perform the simulation using the program. Notably, the chief complaints presented in the simulated clinical case were chest pain and dyspnea, with pulmonary embolism (PE) being the correct final diagnosis. Postsimulation, the students retook a multiple-choice question test, identical to the presimulation test, which was used to measure the changes in the number of right answers. Results were used as a proxy for the students’ short-term knowledge acquisition. During the entire test period, students were not permitted to talk amongst themselves, consult written material, or use cell phones or similar devices. As shown in Multimedia Appendix 2, examples of core questions are questions 3 and 4. Given that the Hepius clinical case dealt with PE, question 3 was asking about the most common physical sign associated with PE (ie, tachycardia), whereas question 4 addressed the diagnostic relevance of low D-dimer plasma levels in excluding PE diagnosis, being that such a blood test was characterized by high negative predictive values. Both are crucial aspects of PE diagnosis and were addressed during the Hepius clinical case by expecting the student to look for these diagnostic factors when performing physical examinations and requesting medical tests, and to identify the correct relationship between these and the PE diagnostic hypothesis during the binary analysis. The remaining noncore questions dealt with issues presented and discussed during the semester’s classes, as it was for PE, but not explicitly dealt with in the simulated clinical case. The aim of the noncore questions was to assess students’ overall knowledge about the topics learned during half of the academic year; the aim was also to discriminate whether possible variations between pre- and postsimulation test scores were only related to knowledge that could be acquired through the simulated clinical case or, on the contrary, whether they were the result of a more generalized effect (eg, repeated-testing effect) [54,55].

Data are expressed as mean (SD). The Student *t* test for paired observations was used to evaluate, in each individual, the changes in the achieved scores before and after the simulation. Differences were considered significant at values of *P*<.05. Prism, version 8 (GraphPad Software), was used for statistical analyses. 

## Results

### Overview

Hepius permits the creation of simulated clinical cases by human tutors and their execution by students. The creator of a simulated clinical case (ie, the tutor in charge) is responsible for creating a reference list containing all the clinically relevant information in the form of diagnostic factors (eg, body temperature = 39 °C), reasonable diagnostic hypotheses (eg, pneumonia and PE), the conceptual relationship between diagnostic factors and diagnostic hypotheses, and the correct final diagnosis. Further details on the creation of a simulation are provided in Multimedia Appendix 3.

### Simulation of Clinical Cases With Interaction in Natural Language

**Figure 3:**
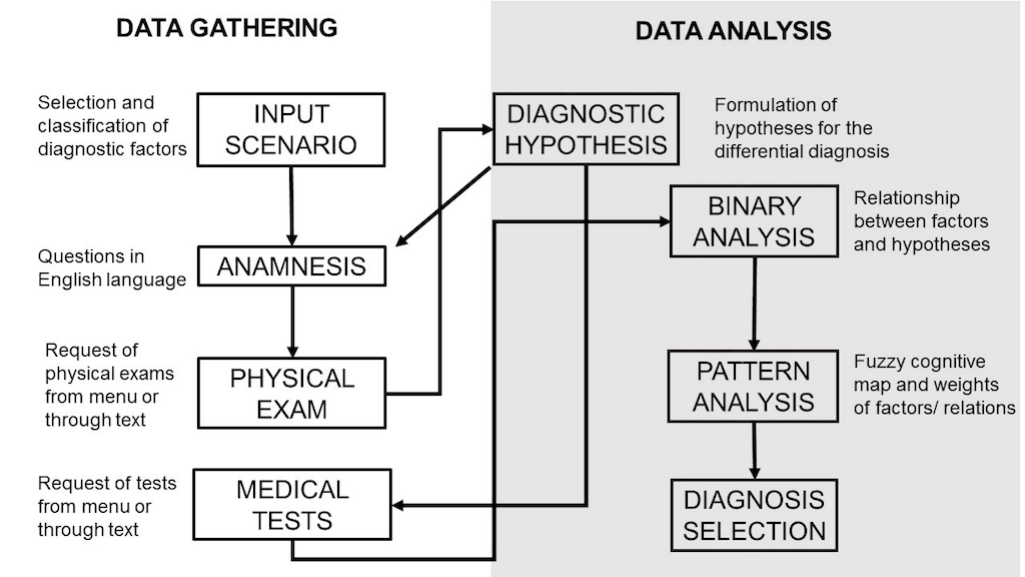
Hepius' flow of use. The flowchart summarizes Hepius' structure and the diagnostic pathway that the student must follow to achieve the final diagnosis. Data gathering deals with the collection of anamnestic, physical, and instrumental data suitable for formulating likely diagnostic hypotheses. Data analysis refers to the differential diagnosis process. During data analysis, the student is asked to generate a diagnostic hypothesis by reasoning on the relationship between the gathered information and the single hypothesized diagnosis. This process is obtained by the binary analysis and the pattern analysis. This should train the learner to avoid ordering unnecessary tests. Selection of the final diagnosis ends the simulation.

The simulation of a clinical case with Hepius requires students to perform multiple actions that can be classified as either data gathering activities or data analysis activities (see Figure 3). *Data gathering* activities consist of obtaining diagnostic factors from the virtual patient through (1) examination of the patient’s health records (ie, the input scenario), (2) anamnesis, (3) a physical exam, and (4) medical test requests. *Data analysis* activities include (1) generating diagnostic hypotheses, (2) establishing causal links between diagnostic factors and diagnostic hypotheses (ie, binary analysis), and (3) estimating the magnitude of these links (ie, pattern analysis). Importantly, Hepius lets the student freely move back and forth within all sections of the simulation, allowing for clinical case reassessment.

In *data gathering* activities, the student has to collect all diagnostic factors that are potentially relevant for the final diagnosis. This is obtained by student-software interaction in natural language rather than by selecting a question or action from a predetermined list. The NLP algorithm then matches the student’s anamnestic question with the most semantically similar reference question and provides its related answer. Natural language interaction is also available when a student performs the physical exam and asks for medical tests.

In the *data analysis* phase, the student works with the collected diagnostic factors to reach a final diagnosis. First, the student creates a differential diagnosis by writing her or his diagnostic hypotheses in natural language. Then, the NLP algorithm matches the student’s diagnostic hypothesis to the semantically closest disease present in the SNOMED ontology. If the matched disease is present in the reference list, then the diagnostic hypothesis is considered correct and is included as part of the student’s differential diagnosis. Once the student deems the differential diagnosis to be complete, the *binary analysis* can be performed (see Table 1). A table is automatically generated, listing the diagnostic factors (rows) and the diagnostic hypotheses (columns) identified thus far, in which the student is expected to outline whether each diagnostic factor increases, decreases, or does not affect the probability that the considered diagnostic hypothesis is the correct one.

**Table 1:** Example of the binary analysis process.

Diagnostic factor^a^ name	Diagnostic factor value	Diagnostic hypothesis^a^	
		Pharyngitis	Pneumonia
Body temperature	38.5 °C	I	I
Pharynx inspection	No pharyngeal erythema	D	N
Chest x-ray	Lobar consolidation	N	I

^a^The diagnostic factors and the diagnostic hypotheses are automatically added to rows and columns, respectively, for *binary analysis*. By selecting the boxes, the student actively chooses whether each diagnostic factor increases (I), decreases (D), or does not affect (N) the probability that the considered hypothesis will be the final diagnosis.

**Figure 4:**
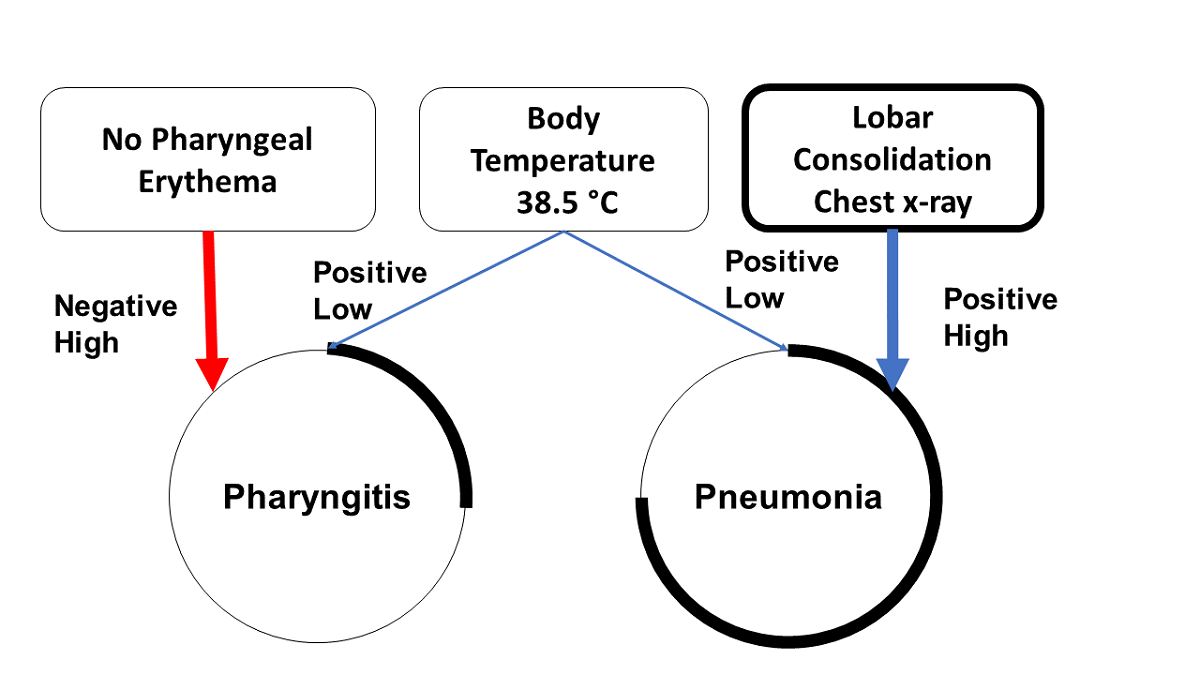
Schematic overview of the pattern analysis process. Should the diagnostic factor increase the probability of the chosen diagnostic hypothesis, then the positive likelihood of such a relationship is represented by a connecting blue line. If a diagnostic factor is thought to decrease the likelihood of the diagnostic hypothesis, then the connecting line is depicted in red. When the diagnostic factor does not affect the diagnostic hypothesis, no connecting line is drawn. In addition, the student is asked to weigh the relevance of the diagnostic factors in relation to the hypothesized diagnoses. This is automatically translated into a graphic representation with an increase (positive) or decrease (negative) of the thickness of the connecting lines. In the example in the image, the presence of lobar consolidations on the chest x-ray was highly suggestive of pneumonia (positive high). Therefore, the thickness of the connecting line becomes wider. The circumference of the diagnostic hypothesis node was related to the probability that the chosen diagnosis was correct. As the probability of diagnosis increased, the portion of the highlighted circumference increased as well.

In the *pattern analysis*, the student can visualize and weigh the relationships among diagnostic factors and diagnostic hypotheses previously established during the *binary analysis*; see Figure 4 for further details. Once the student is satisfied with the analysis of the information previously gathered, the simulation can be ended by selecting the diagnostic hypothesis that is deemed to be correct.

### Intelligent Tutoring System

The ITS tracks all the student actions and provides real-time step-by-step feedback over the simulation’s entire execution. For instance, if the student asks for a medical test that is absent in that clinical case reference list, he or she receives feedback stating that an inappropriate exam was asked for. As another example, should the diagnostic hypothesis made by the student (eg, pneumonia) be too general compared to the one in the reference list (eg, interstitial pneumonia), then feedback is given stating that the student should be more specific in generating the hypothesis. An exhaustive list of possible feedback is provided in Multimedia Appendix 4.

Furthermore, at the end of the simulation, the ITS provides feedback summarizing the diagnostic hypotheses in which the student has made more mistakes when addressing the binary analysis. In addition, links to the UpToDate topics related to these diagnostic hypotheses are given [56].

Moreover, the ITS logs all student actions, enabling post hoc learner analytics. In a related article currently under peer review [57], the possible applications of learner analytics are described in detail.

### Short-term Learning Test Results

**Figure 5:**
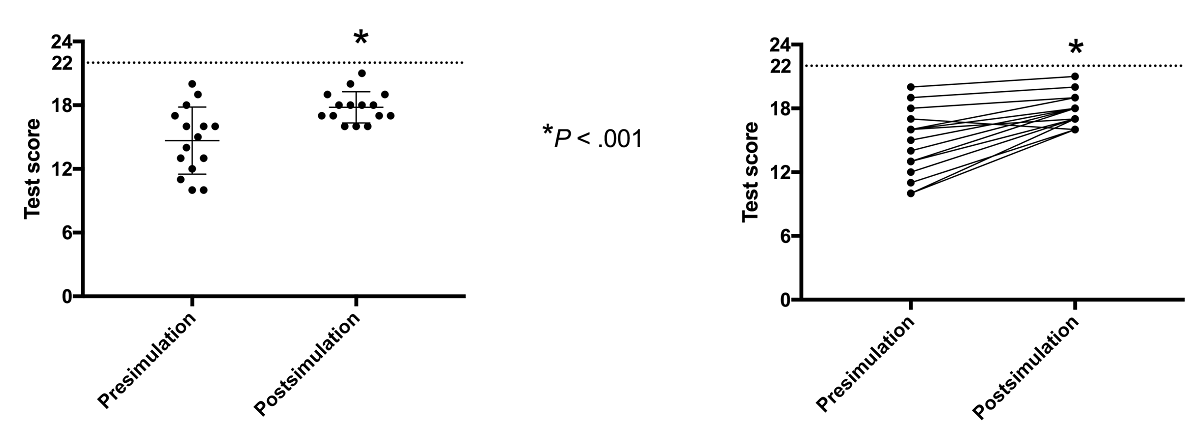
Overall pre- and postsimulation performance. Notice the significant improvement of the overall test score average after the use of Hepius (left-hand graph). Students’ individual performances are shown in the right-hand graph.

A significant improvement was found in the mean postsimulation overall test score compared to the presimulation overall test score (mean 17.8, SD 1.48, vs mean 14.6, SD 3.15, respectively; *P*<.001) (see Figure 5). Students’ individual performances are shown in the right-hand graph of Figure 5. Only one subject’s performance worsened after the simulation.

**Figure 6:**
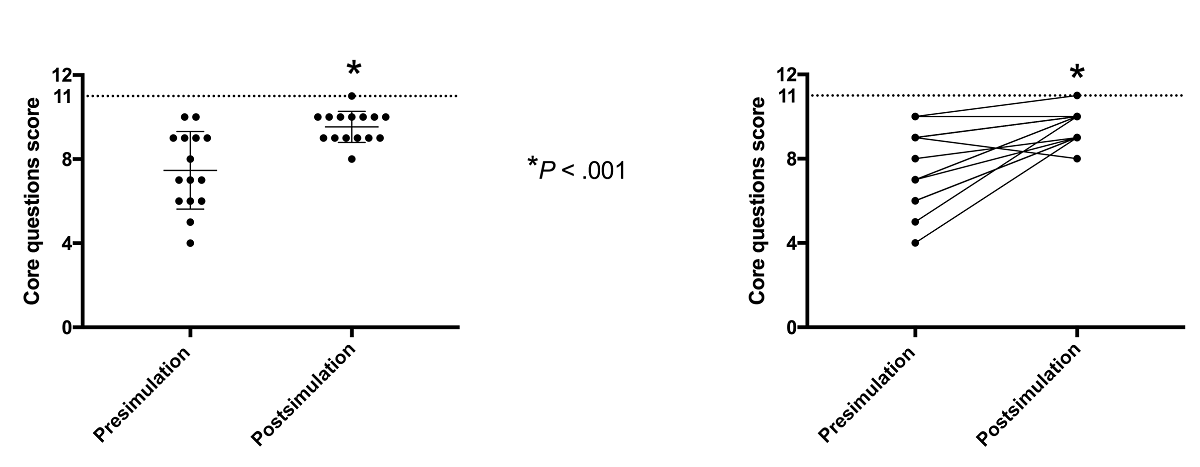
Pre- and postsimulation performance of core questions. The dashed horizontal line indicates the maximal reachable score. Scores are based on 15 students. A significant improvement in the mean score of core questions was observed from pre- to postsimulation tests (left-hand graph). Individual performances are displayed in the right-hand graph.

There was a significant improvement in mean score for *core* questions from pre- to postsimulation (mean 7.46, SD 1.84, vs mean 9.53, SD 0.74, respectively; *P*<.001) (see Figure 6). Notably, out of the 15 students, 13 (87%) improved their *core* question scores from pre- to postsimulation. One student had no change and one obtained a lower score (see Figure 6, right-hand graph).

## Discussion

In this paper, Hepius’ most important features and the preliminary results obtained by its use in a medical undergraduate class are presented. Interaction in natural language and intelligent tutoring are the most important features of the program and are hereafter discussed.

### Virtual Patient Simulators and Natural Language Processing

VPS may play an important role in medical education, particularly in training users in clinical diagnostic reasoning [58]. In the vast majority of VPSs, the interaction between the user and the simulated patient occurs by means of menus and the selection of predefined items [19,59]. The simulator recently developed by the New England Journal of Medicine Group [60] is such an example. It is aimed at training experienced doctors in facing COVID-19 cases that evolve over time according to the user’s diagnostic and therapeutic interventions, which are selected from a predefined list of possibilities. Conversely, Hepius, which is specifically designed for undergraduate medical students, allows interaction through free text in the English language. We assumed that this type of automated interaction might better mirror real-life doctor-patient communication, thus increasing clinical simulation accuracy as previously suggested [22]. Furthermore, the absence of drop-down menus to select the most appropriate action highlights an important educational issue: students have to actively think about questions without getting hints by choosing prepackaged options. The same reasoning could be applied to diagnostic hypothesis generation.

Notably, a potential limitation of NLP techniques may be related to the low accuracy in interpreting questions. This can distract students from the focus of the task, as suggested in 2009 by Cook et al [10]. Nowadays, performance of the newest NLP algorithms has reached an accuracy as high as 95%, thus limiting the risk of users’ frustration for not having their questions understood by the simulator [22].

### Intelligent Tutoring System

ITSs are programs aimed at providing immediate and customized instruction or feedback to learners, without interference from a human teacher [61]. These programs have been proven to be effective as teaching tools within different educational fields [12,13]. However, there are few studies about their use in the medical context. One of these is ReportTutor [62], which is an ITS aimed at helping pathology trainees to write correct biopsy reports in English natural language. Its tutoring activity stems from its capability to identify inaccuracies or missing features within the report and to give appropriate feedback to the trainees. Interestingly, ReportTutor shares NLP techniques with Hepius; however, those of ReportTutor are not devoted to mimicking the doctor-patient interaction.

Hepius integrates the key ITS concepts of *inner loop* (ie, step-by-step feedback and hints during the execution of the learning unit) and *outer loop* (ie, indications of what is the optimal next learning step) [49]. Inner loop feedback is given whenever a student performs an action. For example, if during the binary analysis the student wrongly states that the diagnostic factor *fever* decreases the likelihood of the patient having the diagnostic hypothesis *pneumonia*, then Hepius provides feedback indicating the correct relationship between these two factors. This type of feedback is important not only because it directly fosters learning but also because it allows students to complete their simulation, guiding them throughout the case. Outer loop feedback is instead given at the end of a simulation, according to the overall performance of the student. For example, if a user consistently makes mistakes in matching diagnostic factors to the diagnostic hypothesis *pneumonia*, the ITS recommends that the student review that specific topic by providing her or him with a link to the related UpToDate section. This type of automated feedback directly addresses weaknesses in the student’s knowledge and provides him or her with suggestions on how to correct their mistakes.

### Hepius as a Possible Didactical Tool for Clinical Diagnostic Reasoning

Hepius has been developed as a VPS with the aim of providing an automated training tool for clinical diagnostic reasoning. Clinical reasoning combines *intuitive thinking* (ie, heuristic thinking) and *analytical thinking*. Experienced doctors tend to apply heuristic thinking to an ordinary clinical case and revert to analytical thinking when the case is rare or complex. On the other hand, less experienced physicians mainly rely on analytical thinking [63].

Hepius has been developed to target undergraduate medical students in order to train them in analytical thinking. This mental process is applied, for instance, during the binary analysis, where the student is asked to disclose the causal relationship between each single diagnostic factor and diagnostic hypothesis. In addition, through the pattern analysis, Hepius provides the student with the possibility of visually addressing the relationships between diseases and clinical findings, in a process similar to conceptual maps [64]. Overall, these analytical exercises are expected to help students enhance their diagnostic skills and medical knowledge, although no robust evidence is presently available, except for our preliminary findings. These shall be briefly discussed below.

The capability of Hepius to enhance medical knowledge in the short term was preliminarily evaluated among 15 students attending their fifth year at the Humanitas University Medical School. They completed an identical test, composed of multiple-choice questions, before and after the clinical case simulation by Hepius. We hypothesized that, in such a way, the test would provide proper insight into the potential changes in students’ knowledge on the specific issue dealt with during the simulation (ie, PE). In keeping with previous reports highlighting the educational capabilities of VPSs [10,14], in this study, Hepius use resulted in an increase in the performance scores of almost all the students. This was the case for the students who had good baseline performance as well as for those whose initial performance was poor. Taken together, these findings suggest that, in the short term, Hepius might act as a didactical tool.

However, in spite of its promising features, it is important to stress that Hepius cannot fully replace a skilled human tutor working one on one with a learner [65]. Instead, in keeping with a *blended* approach, it is intended to be used as a classroom assistant as well as a tool for distance learning. Indeed, as with any VPS, Hepius allows for proper social distancing; therefore, it is potentially useful in overcoming the didactical problem regarding the temporary inability to attend clinical facilities in the setting of the COVID-19 outbreak.

### Limitations

As with any automated didactical tool, students’ performance using Hepius is characterized by a learning curve, and its optimized use requires initial tutoring. This is presently provided via a video tutorial and should be refined by teachers through ad hoc online lectures, in accordance with the concept of orchestration of intelligent learning environments [15,66].

Accuracy of the diagnostic hypothesis generation module has not been estimated due to the lack of a comprehensive test set. Also, we have not attempted to use language modeling or semantic similarity algorithms based on a deep learning algorithm approach. Both activities are objectives for future work. Finally, the short-term learning test has been carried out among a small number of students and using a limited pool of questions. Thus, our findings should be regarded as preliminary results that must be confirmed in future studies and further validated on larger cohorts.

### Conclusions

Shortage of human resources, increasing educational costs, and the need to keep social distances in response to the COVID-19 worldwide outbreak have prompted the necessity of automated clinical training methods designed for distance learning. We have developed a VPS named Hepius that, by natural language interaction and an ITS component, might help students to improve their clinical diagnostic reasoning skills without necessarily requiring the presence of human tutors or the need for the student to be at the bedside of a real patient. Implementation of additional features, such as therapy and patient management modules, can be pursued to make Hepius suitable for application in postgraduate residency programs and continuing medical education.

As a preliminary assessment of its educational impact, we found that the use of Hepius may enhance students’ short-term knowledge. Ad hoc studies using larger populations are needed to confirm this result and to investigate Hepius’ actual long-term didactical capability.

## Appendix

Multimedia Appendix 1 Natural language matchers.

Multimedia Appendix 2 Multiple-choice question test.

Multimedia Appendix 3 Creation of a simulation.

Multimedia Appendix 4 Inner-loop feedback.

## Abbreviations

CEO: Chief Executive Officer
HMM: hidden Markov model
ITS: intelligent tutoring system
LSTM: long short-term memory
NLP: natural language processing
NLTK: Natural Language Toolkit
PE: pulmonary embolism
PyMedTermino: Medical Terminologies for Python
SLSTM: Siamese long short-term memory
SNOMED: Systematized Nomenclature of Medicine
SNOMED CT: Systematized Nomenclature of Medicine Clinical Terms
SQL: Structured Query Language
VPS: virtual patient simulator
