# Supplementary material for: A Natural Language Processing–Based Virtual Patient Simulator and Intelligent Tutoring System for the Clinical Diagnostic Process: Simulator Development and Case Study
Source: JMIR Med Inform. 2021 Apr 9;9(4):e24073. doi: 10.2196/24073 (PMC8041050; doi:10.2196/24073)
Supplement: Multimedia Appendix 1 [file medinform_v9i4e24073_app1.doc]

Table S1. Performance of anamnestic question matcher

| **Simulation name** | **Percentage of rank**  **1 matches (%)** | | **Percentage of rank**  **in-first-three matches (%)** |
| --- | --- | --- | --- |
| Mr Ad | 87.40 | | 92.44 |
| Mr Ar | 80.65 | | 85.48 |
| Mr M | 72.64 | 79.25 | |
| Mr X | 87.01 | 90.91 | |
| Ms Ea | 63.40 | 70.71 | |
| Ms Er | 90.28 | 91.67 | |

The rank 1 column shows the percentage of test question pairs for which the algorithm has identified the correct matching questions and returned it with the highest score. The rank in first three matches column shows the percentage of test question pairs for which the correct matching question was returned with one of the top three scores. Our goal was to exceed 70% in rank 1 matches and 80% in rank 3 matches. Overall, the results are satisfactory with the only exception of Ms Ea simulation.

Whenever the student formulates a diagnostic hypothesis, *Hepius* enables the identification of the correct one among all the hypothesized diagnoses inserted by the author.

To cope with the problem of *diagnostic hypotheses matching,* two main features were embedded into *Hepius,* as follows:

1. When the teacher sets the clinical case (see Appendix 2 – Creation of a simulation), an *ad hoc* developed NLP algorithm checks each proposed DH into SNOMED CT ontology [1] in the attempt to optimize the matching. It is up to the author to decide whether the program has found the best matching.
2. When students formulate their own hypotheses, another *ad hoc* developed NLP algorithm checks whether there are equivalent matches among the author’s hypotheses.

Reference

1. Bodenreider O., Cornet R. and Vreeman D.J. Recent Developments in Clinical Terminologies — SNOMED CT, LOINC, and RxNorm. Yearb Med Inform. 2018 Aug; 27(1): 129–139. Published online 2018 Aug 29. doi: 10.1055/s-0038-1667077. PMCID: PMC6115234. PMID: 30157516.
