# Supplementary material for: A Natural Language Processing–Based Virtual Patient Simulator and Intelligent Tutoring System for the Clinical Diagnostic Process: Simulator Development and Case Study
Source: JMIR Med Inform. 2021 Apr 9;9(4):e24073. doi: 10.2196/24073 (PMC8041050; doi:10.2196/24073)
Supplement: Multimedia Appendix 2 [file medinform_v9i4e24073_app2.docx]

Appendix 2. Multiple-choice question test. The test performed by the students to assess short-term knowledge changes was composed of 22 multiple-choice questions concerning the diagnostic approach to a patient presenting with chest pain or dyspnea. Eleven of these questions (“core questions”) had been specifically designed to evaluate knowledge that could be acquired directly by performing the simulation with Hepius. Specifically, core questions were centered on the issue of pulmonary embolism, that was the correct final diagnosis of the simulated clinical case. Hereafter we report 2 core questions, marked by an asterisk, and 2 non-core questions taken by the original test. Correct answers are in bold.

1) A patient presents to the emergency room complaining of shortness of breath of sudden onset, weakness but no chest pain. ECG shows nonspecific ST-T changes. In which of the following conditions could a painless myocardial infarction most likely occur:

1. Premenopausal women
2. **Elderly diabetic patients**
3. Myocardial infarction of the inferior cardiac wall
4. Patients with advanced coronary artery disease on multiple medications
5. All the above

2) A 28-year-old male of Moroccan origins presents to the outpatient clinic with intermittent symptoms of dyspnea on exertion, palpitations, and cough occasionally productive of blood. On cardiac auscultation, a low-pitched diastolic rumbling murmur is faintly heard toward the apex. The origin of the patient’s problem probably relates to:

- 1. **Rheumatic fever**
  2. Long standing hypertension
  3. Silent MI within the past year
  4. Congenital origin
  5. Lung cancer

*3) Which is the most common ECG finding in a patient with pulmonary embolism:

1. **Sinus tachycardia**
2. S1Q3T3
3. Signs of right ventricular strain
4. ST elevation from V2 to V6
5. Pathologic Q waves in the inferior leads

*4) A 32-year-old previously healthy woman comes to the emergency room because of sudden shortness of breath and right sided pleuritic chest pain. She has been using oral contraceptive pills for the past 6 years. On physical exam, she is tachypnoic, normotensive and tachycardic. Which of the following results would be most useful in excluding the suspected diagnosis?

1. Normal cardiopulmonary exam
2. Normal chest x ray
3. Sinus tachycardia on ECG
4. **Normal D-dimer plasma levels**
5. Increased troponin plasma levels
