# Supplementary material for: A Natural Language Processing–Based Virtual Patient Simulator and Intelligent Tutoring System for the Clinical Diagnostic Process: Simulator Development and Case Study
Source: JMIR Med Inform. 2021 Apr 9;9(4):e24073. doi: 10.2196/24073 (PMC8041050; doi:10.2196/24073)
Supplement: Multimedia Appendix 3 [file medinform_v9i4e24073_app3.doc]

***Creation of a simulation***

Authorized users can create new simulations that will be added to the *Hepius* library. The suggested first step is to set the differential diagnoses around which the clinical case will be built by listing all the clinically reasonable DHs that the student is expected to take into consideration according to his/her specific level of knowledge. Then, for each component of the data gathering section, the creator should add all the DFs that are believed to be useful in managing that specific simulated patient. In addition, some DFs in each section are set as default so that, unless otherwise specified by the creator, they will be present in every simulation. For example, the question “Do you have any allergies?” will be present by default in every simulation with the answer “No”. As all the DHs and DFs are being added to the new simulation, the creator can proceed towards the last steps. These involve defining the correct values for the binary analysis and determining the correct final diagnosis. Presently, the time required to create a new simulation from scratch is about 3-4 hours.

Importantly, by means of the *“cloning”* feature, teachers have the possibility to create new simulations from existing ones by simply modifying the results of a few DFs (e.g., the results of a radiology test), thereby entirely changing the clinical scenario and final diagnosis. This process requires just few minutes per clone and may substantially expands the *Hepius* library.

Of note, the creation of a simulation by the students may also be used as an educational tool to improve their disease knowledge, in keeping with the educational concept *learning by teaching* [1].

Reference

1. Leelawong, K., & Biswas, G. Designing learning by teaching agents: The Betty’s brain system. International Journal of Artificial Intelligence in Education, 2008;18(3), 181–208.
