# Supplementary material for: A Natural Language Processing–Based Virtual Patient Simulator and Intelligent Tutoring System for the Clinical Diagnostic Process: Simulator Development and Case Study
Source: JMIR Med Inform. 2021 Apr 9;9(4):e24073. doi: 10.2196/24073 (PMC8041050; doi:10.2196/24073)
Supplement: Multimedia Appendix 4 [file medinform_v9i4e24073_app4.docx]

Appendix 4. Inner-loop feedbacks. Hepius provides a real-time feedback for every action the student performs throughout the simulation. In the following tables is provided a comprehensive list of all the feedbacks Hepius can provide, together with the corresponding action that elicits the feedback. Each table represents a different section of the simulation.

Table S1. Feedbacks related to the anamnesis.

| **Student action** | **Feedback** |
| --- | --- |
| Students asks a question that is present in the reference list | *“Ok”* |
| Student asks a question that is not present in the reference list | *“The question you have asked is not relevant for this clinical case”* |
| Student makes a typing error | *“It seems that your question is not well typed”* |
| Student asks a question that is equivalent to a question he already asked | *“You have already asked this question”* |

Table S2. Feedbacks related to the physical exam.

| **Student action** | **Feedback** |
| --- | --- |
| Student inserts an appropriate exam | *“Correct Exam”* |
| Student inserts a non-existing exam | *“Please insert a valid physical exam”* |
| Student inserts a poorly specified exam | *“Your specification is not complete. Please specify an exam object”* |
| Student does not specify appropriately the imaging exam requested | *“Your specification is not complete. You did not insert a valid anatomical region”* |
| Student inserts a previously performed exam | *“You have already performed this physical exam”* |

Table S3. Feedbacks related to medical tests requests.

| **Student action** | **Feedback** |
| --- | --- |
| Student requests an appropriate exam | *“Good Work”* |
| Student requests a non-relevant exam | *“The exam you have selected is not relevant for this clinical case”* |
| Student requests a non-existing exam | *“Please insert a valid medical test”* |
| Student does not specify appropriately the imaging exam requested | *“Your specification is not complete. You did not insert a valid anatomical region”* |
| Student requests a previously requested exam | *“You have already requested this examination”* |

Table S4. Feedbacks related to diagnostic hypotheses generation.

| **Student action** | **Feedback** |
| --- | --- |
| Student formulates a correct hypothesis | *“Your input is a correct hypothesis. You are on a good track!”* |
| Student over specifies a correct hypothesis | *“Your input is too specific, but it's not so far from the correct hypothesis. Please try again!”* |
| Student poorly specifies a correct hypothesis | *“Your input is generic, but it's not so far from the correct hypothesis. Please try again!”* |
| Student formulates a wrong hypothesis | *“Your hypothesis is not correct”* |
| Student repeats a correct hypothesis | *“You have already included this hypothesis”* |

Table S5. Feedbacks related to the binary analysis.

| **Student action** | **Feedback** |
| --- | --- |
| Student makes a correct causal relationship between a DF and a DH | “Good work!” |
| Student makes a wrong causal relationship between a DF and a DH | “Your analysis, is not correct. Please try again” |
